# 18-month longitudinal SARS COV-2 neutralizing antibody dynamics in haemodialysis patients receiving heterologous 3-dose vaccination (AZD-1222- AZD-1222- BNT162b2) in a lower middle income setting

**DOI:** 10.1186/s12882-024-03599-7

**Published:** 2024-05-22

**Authors:** Ridma Prasadini Karunathilake, Roshan Athula Kumara, Amali Karunathilaka, Abdul Wahid Mohamed Wazil, Nishantha Nanayakkara, Chandana Keerthi Bandara, Rajitha Asanga Abeysekera, Faseeha Noordeen, Indika Bandara Gawarammana, Champa Neelakanthi Ratnatunga

**Affiliations:** 1 Department of Microbiology, Faculty of Medicine, University of Peradeniya, Peradeniya, 20400 Sri Lanka; 2 Nephrology Unit, National Hospital Kandy, Kandy, 20000 Sri Lanka; 3 Department of Medicine, Faculty of Medicine, University of Peradeniya, Peradeniya, 20400 Sri Lanka; 4 Center for Education, Research and Training in Kidney Disease (CERTKiD), University of Peradeniya, Peradeniya, 20400 Sri Lanka

**Keywords:** Haemodialysis, COVID-19, Mixed-vaccination, Seroconversion, Neutralizing antibody levels, Longitudinal follow-up

## Abstract

**Background:**

Patients with chronic kidney disease on haemodialysis (HD) were given priority COVID-19 vaccination due to increased disease risk. The immune response to COVID-19 vaccination in patients on HD was diminished compared to healthy individuals in 2-dose studies. This study aimed to evaluate seroconversion rate, neutralizing antibody (nAB) levels and longitudinal antibody dynamics to 3-dose heterologous vaccination against COVID-19 in a cohort of HD patients compared to healthy controls and assess patient factors associated with antibody levels.

**Methods:**

This study was a case–control longitudinal evaluation of nAB dynamics in 74 HD patients compared to 37 healthy controls in a low/middle income setting. Corresponding samples were obtained from the two cohorts at time-points (TP) 1–1-month post 2nd dose of AZD1222 vaccine, TP2- 4 months post 2nd dose, TP4- 2 weeks post 3rd dose with BNT162b2 vaccine, TP5-5 months post 3rd dose and TP6-12 months post 3rd dose. Additional data is available at TP0- pre 2nd dose and TP3- 6 months post 2nd dose in HC and HD cohorts respectively. Anti-SARS-CoV-2 nAB were detected using Genscript cPassTM pseudoviral neutralization kit. Demographic and clinical details were obtained using an interviewer administered questionnaire.

**Results:**

Cohorts were gender matched while mean age of the HD cohort was 54.1yrs (vs HCs mean age, 42.6yrs, *p* < 0.05). Percentage seroconverted and mean/median antibody level (MAB) in the HD cohort vs HCs at each sampling point were, TP1-83.7% vs 100% (*p *< 0.05), MAB-450 IU/ml vs 1940 IU/ml (*p* < 0.0001); TP2-71.4% vs 100%, (*p* < 0.001), MAB- 235 IU/ml vs 453 IU/ml, (*p* < 0.05); TP4-95.2% vs 100% (*p* > 0.05), MAB-1029 IU/ml vs 1538 IU/ml (*p* < 0.0001); TP5-100% vs 100%, MAB-1542 IU/ml vs 1741IU/ml (*p* > 0.05); TP6-100% vs 100%, MAB-1961 IU/ml vs 2911 IU/ml (*p *> 0.05). At TP2, patients aged < 60 years (*p* < 0.001) were associated with maintaining seropositivity compared to patients > 60 years.

**Conclusion:**

Two dose vaccination of haemodialysis patients provided poor nAB levels which improved markedly following 3rd dose vaccination, the effect of which was long- lasting with high nAB levels in both patients and controls detectable at 1 year follow-up.

**Supplementary Information:**

The online version contains supplementary material available at 10.1186/s12882-024-03599-7.

## Background

Patients on dialysis had a 16–32% increased risk of fatality from COVID-19 compared to the general population in 2020–2021 [1–8] with in-centre dialysis carrying additional risk with compared to home dialysis [1, 8–10]. Correction for comorbidities and demographic factors still showed a four-fold increase in mortality among dialysis patients, highlighting their vulnerability [11, 12], justifying their priority vaccination status.

The muted vaccine responses in haemodialysis (HD) patients to vaccines [13–15] necessitating additional primary series dosing, hinted that standard two-dose COVID-19 vaccination may not be sufficient in these patients. Confirming this, multiple studies demonstrated that HD patients developed poor two-dose immunity compared to the healthy controls [16–22]. Meta-analysis of 38 studies on two-dose immunogenicity in HD patients showed 85.1% seroconversion compared to 97.4% in healthy controls [17]. Vaccine platform also affected seroconversion with high rates (> 96%) observed for mRNA vaccines but significantly lower rates (67%) for the Ad26.COV2.S vaccine [23] and inactivated vaccine (80%) [24]. HD patients had rapid antibody decay and by 6 months, 20% had no detectable antibodies [25, 26]. Factors contributing to improved longevity of response included serum albumin level, anti-HBs antibody level > 10 IU/ml and specific races [26].

In October 2021, the Strategic Advisory Group of Experts (SAGE) of the World Health Organization (WHO) issued interim guidelines that all immune compromised patients (including long-term dialysis) receive a three-dose primary series where possible [27]. Recent studies confirm the improved response to third dose of mRNA vaccine in achieving sustained seroconversion and antibody levels in HD patients [16, 20, 28–31]. Limited evidence exists on the efficacy of heterologous/mixed vaccine combinations in HD patients on maintaining long term immunity.

While Sri Lanka, a lower middle income South Asian country, with a population of 21 million, endemic for chronic kidney disease of unknown aetiology (CKDu) with a national haemodialysis population of over 3500 patients [32], followed similar guidelines, practicalities of vaccine availability resulted in the use of a heterologous combination of two vaccines in HD patients. The same combination was administered to health care workers (HCWs) at the same times, providing an ideal control group. The objective of this study was to evaluate seroconversion rate, neutralizing antibody (nAB) level and longitudinal antibody dynamics to three-dose mixed vaccination against COVID-19 in a cohort of HD patients compared to healthy controls and assess patient factors associated with antibody levels, up to one year after third dose, as nAB levels correlate with protection from infection which is a priority in these individuals. We analyzed breakthrough SARS CoV-2 infection as Sri Lanka went through waves of the virus variants, as well as factors associated with seroconversion and high nAB levels, and comparing vaccine associated adverse events (VAAE) in these two groups. We hypothesized that HD patients would show lower seroconversion rates and lower / short lasting antibody responses compared to HCs in this setting.

## Methods

### Ethical considerations

This study was approved by the ethical review committee of the Faculty of Medicine, University of Peradeniya (protocols EC/2021/13 and EC/2021/14). Informed written consent was obtained from all participants prior to interview and sample collection.

### Study population

This longitudinal case–control study was conducted from May 2021 at the beginning of national COVID-19 vaccination programme to December 2022, 1 year following the administration of the third dose. Initially, 74 HD patients who attend the Nephrology units of National Hospital Kandy and Teaching Hospital Peradeniya, both large tertiary care centers in the Central Province of Sri Lanka, were recruited to the study. As the control cohort, 37 healthy healthcare workers were recruited. Inclusion criteria for patients were patients > 18 years, on HD who received two- dose vaccination, and health care workers about to complete two dose vaccination. Individuals who refused vaccination or with other causes of immunosuppression, including medication, malignancy, were excluded.

**Fig. 1:**
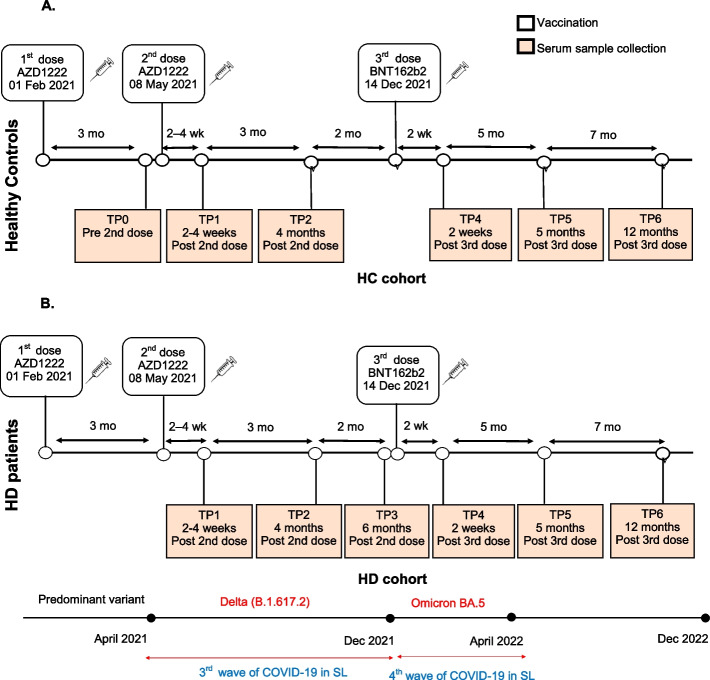
Timeline of vaccination and sampling timepoints. The timeline shows the timing of vaccination and sampling time points (TP) of the healthy controls (HCs) (**A**) and haemodialysis (HD) patients (**B**)

The two groups of HD (case) and HC (control) were given two doses of Oxford Astrazeneca -AZD1222 (ChAdOx1) three months apart, and subsequently a 3rd dose of BNT162b2 was given 6 months later (Fig. 1A and B). Serum sample collection was done at seven time points as shown in Fig. 1: Pre second dose (TP0), 2 weeks post second dose (TP1), 4 months post second dose (TP2), 6 months post second dose (TP3), 2 weeks post third dose (TP4) and 5 months post third dose (TP5) and one year post third dose (TP6). All timepoints (TP) could not be sampled in both groups due to logistic difficulties encountered. A summary of patient and control numbers included at each time point are shown in Additional file 1.

An interviewer administered questionnaire was used to collect demographic data including age and gender, history of COVID-19 including symptoms, or laboratory confirmed infection at each time point, and VAAE related to each dose. In HD patients, duration on dialysis and comorbidities were documented.

Blood samples were obtained at prescribed timepoints. Separated serum samples were stored at -80 °C until testing with GenscriptC Pass™ SARS-CoV-2 neutralization Antibody Detection kit (GenScript USA/ Nanjing GenScript Diagnostics Technology Co., Ltd., Version RUO for US.1.0) according to manufacturer’s instructions [33]. Seroconversion was defined as nAB above 30% inhibition (manufacturer recommended). Results are expressed as % seroconverted (% individuals with nAB levels above cut-off), mean/ median antibody level (MAB) as % inhibition (the midpoint of % inhibition of the positive samples). nAB level in IU/ml (log scaled) was calculated as per WHO guidelines [34] and conversion algorithm for this assay [35] with calculation of geometric mean and SD factor. Samples with > 97.59% inhibition (33/381; 18-HD, 15-HC) required further dilution for accurate conversion to IU/ml. Due to financial constraints, only 4/35 samples were diluted and retested. As true values of all 33 samples could not be obtained, these are plotted as > 3028 IU/ml in Figures and were taken as 3028 IU/ml (equivalent of 97.59% inhibition) for calculations.

### Statistical analysis

Statistical analysis was carried out using GraphPad Prism (8.4.2. 679, 2020). Percentage neutralization was compared using Mann Whitney U (MWU) test and association between categorical variables was evaluated using the chi-square test. Multiple groups were compared with the Kruskal Wallis test and Dunns Post-hoc test for multiple comparisons [36].

## Results

We recruited 74 HD patients (HDs) and 37 HCs. Over 18-month follow-up, sample numbers were limited due to test kit limitations as well as logistic restrictions during the ensuing economic crisis limiting participation. At final sampling, 14 HDs and 16 HCs remained. The number of samples collected and loss of participants at each TP are shown in Additional file 1. At initial sampling, all participants were asymptomatic, had no history of laboratory confirmed COVID-19, or symptoms suggestive of COVID-19 since the start of the pandemic. During follow up, four HCs had laboratory confirmed COVID-19 while none of the HDs tested positive at any time.

### Demographic characteristics

The gender distribution in both cohorts was similar with males comprising 69% (51/74) of the HDs and 48.6% (19/37) in the HCs. (Fishers exact test, *p* > 0.05). Age ranged from 24–80 years (mean 54.1 (95%CI:51.3–56.9) in HDs and 26–59 years (mean 42.6 (95%CI:39.4–45.7)) in HCs (unpaired t test, *p* < 0.0001).

HD patients had a mean HD duration of 29.7 months (SD ± 18.55) (range 4 months to > 6 years). Dialysis vintage and comorbidity distribution are shown in Table 1. Hypertension (81%) and diabetes (40.5%) were the most common comorbidities in this group.

**Table 1:** HD (vs HC) cohort characteristics

Characteristic	Value/ Frequency
**Age﻿**	
Mean age	54.1 years (95%CI:51.3–56.9) vs HC: 42.6 years (95%CI:39.4–45.7)
Age range	24–80 years vs HC: 26–59 years
**Sex**	
Male	69% (51/74) vs HC 48.6% (19/37)
Duration on dialysis (DD)	
Mean DD	29.7 months (SD ± 18.55)
Range DD	4—81 months
< = 12 months	27% (20/74)
13–24 months	23% (17/74)
25–36 months	19% (14/74)
> 36 months	31% (23/74)
Comorbidities	
Diabetes mellitus	40.5% (30/74)
Hypertension	81% (60/74)
Ischemic heart disease	17.6% (13/74)
Dyslipidemias	24.3% (18/74)
Stroke	0
Glomerulonephritis	0

### Longitudinal antibody dynamics following two doses of Oxford Astrazeneca -AZD1222 (ChAdOx1)

**Fig. 2:**
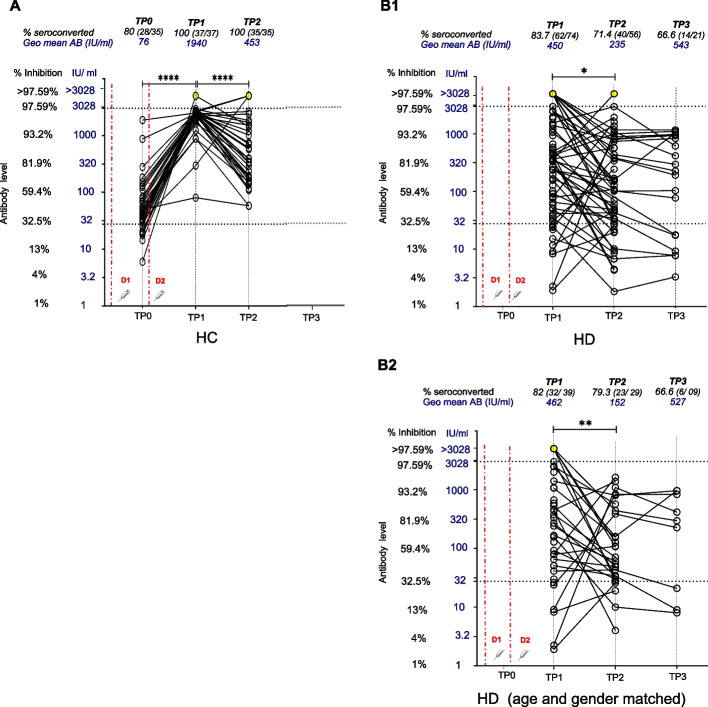
Longitudinal antibody dynamics following two doses of AZD1222. Neutralizing antibodies level as percentage inhibition and IU/ml developed against SARS CoV-2 following vaccination with 2 doses of Oxford Astrazeneca -AZD1222 (ChAdOx1) were plotted at each time point. **A**) shows the antibody dynamics of the HC cohort at the time points of pre second dose (TP0), 2 weeks following second dose (TP1) and 4 months following second dose (TP2). TP3 samples could not be collected in HCs for logistic reasons. **B1**) shows the antibody dynamics of the total HD patient cohort which was gender-matched HD cohort at the time points of TP1, TP2 and TP3 (6 months following 2nd dose) **B2**) shows the antibody dynamics of an age and gender-matched HD subgroup analysis at the time points of TP1, TP2 and TP3. Dot plot shows the international unit (IU/ml) values (primary y axis) while secondary y axis shows the equivalent % neutralization. Seropositivity is designated at > 30% (28 IU/ml) (open circles) (manufacturers standard). Yellow circles plotted along the level of > 3028 IU/ml (> 97.59% inhibition) indicate the individuals with high antibody levels that required further dilution for precise estimation but could not be tested. Red dashed vertical lines indicate the time points of vaccine administration. (D1– first dose, D2– second dose). Percentage seroconversion rate (number seroconverted/ total number sampled) and geometric mean of antibody level (IU/ml) at each timepoint are shown in above each timepoint in black and blue text respectively. MAB levels between timepoints were compared with the Mann Whitney U (MWU) test with statistical significance denoted by *p* < 0.05-*, *p *< 0.01- **, *p* < 0.001—***, *p* < 0.0001—****

Sampling was done at 2–4 weeks (TP1), 4 months (TP2) and 6 months (TP3) post 2nd dose. While a pre 2nd dose (TP0 – immediate pre-2nd dose) sample was collected in HCs, TP3 samples in HCs and TP0 in HD patients were not collected for logistic reasons. Figure 2A shows antibody dynamics in HCs following 2-dose AZD1222. At TP0, a 80% (28/35) seroconversion rate with moderate MAB level of 45.21% inhibition (IQR:36.8%-63.8%) equivalent to geo.mean 76.17 IU/ml (SD factor-2.7) was seen. Significant improvement in all metrics were seen by TP1, with 100% (37/37) seroconversion (chi sq, *p* < 0.001) whilst MAB level increased significantly to 97% inhibition (IQR:96.5%-97.2%) (MWU, *p* < 0.0001), equivalent to 1940 IU/ml (SD factor-2) (MWU, *p* < 0.0001). At TP2, seropositivity remained at 100%(35/35) though MAB significantly decreased to 84.61% neutralization (IQR:72.1%-95.1%) (MWU, *p *< 0.0001), equivalent to 452.9 IU/ml (SD factor-3.3) (MWU, *p* < 0.0001).

Antibody dynamics in the HD cohort (Fig. 2B1) at TP1 (2-4 weeks post 2^nd^ dose) 83.7% (62/74) seroconversion, with MAB level of 85.7% inhibition (IQR:65.1%-96.2%)/ correlating 450.2 IU/ml (SD factor-4.2) was seen. By TP2, seropositivity dropped significantly to 71.4% (40/56) (chi sq, *p* < 0.05) with MAB dropping significantly to 70.35% inhibition (IQR:48.3%-91.9%) (MWU, *p* < 0.05), equivalent to 235.3 IU/ml (SD factor-4) (MWU, *p* < 0.05). At TP3, seropositivity reduced to 66.7% (14/21) while MAB level showed a slight increase to 90.81% (IQR:75.7%-93.2%), equivalent to 543.4 IU/ml (SD factor-2.2). The increase was not statistically different compared to TP2. A subgroup analysis on HD patients (*n* = 39) who were age and gender matched to HCWs at TP1 and TP2 (Fig. 2B2) showed similar results.

### Effect of heterologous third dose with BNT162b2 in HCs and HD patients

**Fig. 3:**
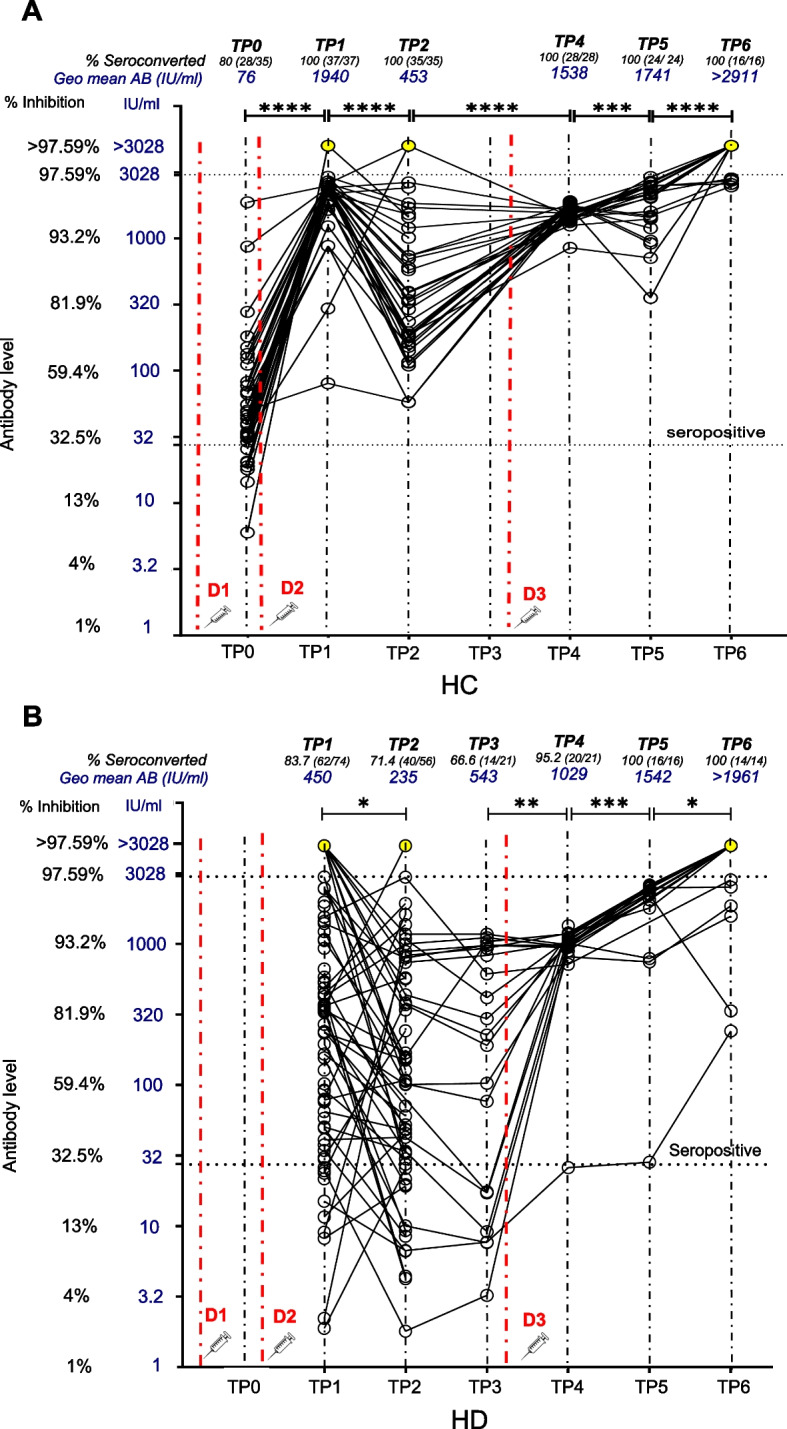
Longitudinal antibody dynamics following two doses of AZD1222 and a third dose of BNT162b2. Level of neutralizing antibodies against SARS CoV-2 following vaccination with two doses of Oxford Astrazeneca -AZD1222 (ChAdOx1) and a third dose of Pfizer-BioNTech (BNT162b2) were plotted at each follow-up time point. Figure 3**A** shows the antibody dynamics of the HC cohort at the time points of pre second dose (TP0), 2 weeks post second dose (TP1), 4 months post second dose (TP2), as well as 2 weeks post third dose (TP4), 5 months post third dose (TP5) and 12 months post third dose (TP6). Figure 3**B** shows the antibody dynamics of the HD cohort at the time points of TP1, TP2, TP3-6 months post second dose, TP4, TP5 and TP6. Dot plot shows the international unit (IU/ml) values (primary y axis) while secondary y axis shows the equivalent % neutralization. Seropositivity is designated at > 30% (28 IU/ml) (open circles) (manufacturers standard). Yellow circles plotted along the level of > 3028 IU/ml (> 97.59% inhibition) indicate the individuals with high antibody levels that required further dilution for precise estimation but could not be tested. Red dashed vertical lines indicate the time points of vaccine administration. (D1– first dose, D2– second dose, D3- third dose) with statistical significance denoted by *p* < 0.05-*, *p* < 0.01- **, *p* < 0.001—***, *p* < 0.0001—****

Two weeks following administration of the BNT162b2 vaccine as the 3rd dose (TP4), HCs maintained a 100% (28/28) seroconversion, while the MAB increased significantly to 95.51% inhibition (MWU, *p* < 0.0001) (IQR:95%-95.8%), / 1538 IU/ml (SD factor:1.17) (MWU, *p* < 0.0001) compared to TP2, as TP3 data was not available for HCs. At TP5 (5 months post 3rd dose), seropositivity remained 100% (24/24) with the MAB significantly increasing to 96.6% inhibition (IQR:95%-97.1%) (MWU, *p* < 0.05) / 1741 IU/ml (SD factor:1.66) (MWU, *p* < 0.05). At TP6 (12 months post 3rd dose), seropositivity remained at 100% (16/16), while MAB increased further to 97.84% inhibition (IQR:97.3–98) (MWU, p < 0.0001), / 2911 IU/ml (SD factor-1.06) (MWU, *p* < 0.0001) as shown in Fig. 3A. In HD patients (Fig. 3B) 3-dose heterologous primary series showed similar results at lower magnitude. At TP4, (2 weeks after 3rd dose), both the seropositivity and MAB significantly increased to 95.2% (20/21) (chi sq, *p* < 0.001) and 93.3% inhibition (SD ± 0.9) (MWU, *p* < 0.005) / 1029 IU/ ml (SD factor:1.15), respectively. At TP5, both seropositivity and MAB rose further to 100% (16/16) (chi sq, *p* < 0.05) and 96.83% inhibition (IQR: 96.2%-97.1%) (MWU, *p* < 0.001) /1542 IU/ml (SD factor:3.1) respectively. At TP6, 12 months post 3rd dose, seropositivity remained 100% (14/14) with MAB significantly increasing (MWU, *p* < 0.05) to 97.75% (IQR:96%-97.9%)/ > 1961 IU/ml (SD factor:2.32).

The loss to follow-up of many patients is noted here. Third dose status was traceable in 51/74 HD patients, of whom 82% (42/51) took the 3rd dose while 18% (9/51) refused. Of those who received the third dose, 50% (21/42) were followed up in the study (at least one timepoint), while 50% were lost to follow-up. Travel restrictions and crises of fuel due to the economic collapse was a major challenge at this time. Post study participant tracing of HD patients lost to follow-up during study period (29 of 53 traced -Additional file 2) showed that while 8/29 did not receive the third dose, only one developed COVID-19 over the subsequent 2 year period.

### Comparison between HCs and HD patients—percentage seroconversion and MAB

A comparison of % seroconverted and MAB level (geometric mean IU/ml and % inhibition) of the HCs and HDs at each TP are shown in Table 2. HDs had a persistently lower seroconversion rate and lower nAB level (both as % inhibition and IU/ml) compared to HCs until TP4- 2 weeks post 3rd dose at which point though seroconversion rates equalize, nAB levels remain significantly lower in HDs Subsequent testing showed comparable nAB levels as well as 100% seroconversion in both cohorts.

**Table 2:** Comparison of seroconversion and MAB in gender-matched cohorts

	**Measure**	**TP1** **2–4 wks post 2nd dose**	**TP2** **4 mo post** **2nd dose**	**TP4** **2 wks post** **3rd dose**	**TP5** **5 mo post** **3rd dose**	**TP6** **12 mo post** **3rd dose**
**HD**	**% Seroconverted** ^***a***^MAB geo.mean (%inhibition)	**83.7%** 450.2 IU/ml (85.70%)	**71.4%** 235.4 IU/ml (70.35%)	**95.2%** 1029 IU/ml (93.30%)	**100%** 1542 IU/ml (96.83%)	**100%** 1961 IU/ml (97.75%)
**HC**	**% Seroconverted** ^***a***^MAB geo.mean (%inhibition)	**100%** 1940 IU/ml (97%)	**100%** 452.9 IU/ml (84.61%)	**100%** 1538 IU/ml (95.51%)	**100%** 1741 IU/ml (96.6%)	**100%** 2911 IU/ml (97.84%)
***P*** **value**	^***b***^***Chi sq***	***P*** **= 0.008**	***P*** **< 0.001**	***P*** **= 0.412**		
	^***c***^*MWU* *(MWU)*	*P* < *0.0001* *(P* < *0.0001)*	*P* < *0.05* *(P* < *0.05)*	*P* < *0.0001* *(P* < *0.0001)*	*P > 0.05* *(P > 0.05)*	*P > 0.05* *(P > 0.05)*

^a^MAB- mean/ median antibody level of the seropositives at each timepoint. Represented as geometric mean (geo.mean) in International Units (IU/ml) and as mean percentage inhibition (% inhibition)

^b^Chi sq test was performed to compare the % seroconverted at each timepoint

^c^Mann Whitney U (MWU) test was performed to compare the MAB levels of seropositives in both International units (IU/ml) and % inhibition. Comparison of % inhibition at each timepoint is shown within brackets. Significance at *p* < 0.05

Sufficient HD patients to perform an age and gender-matched HD subgroup analysis were available at TP1 and TP2. A summary of these results are shown in Table 3 showing a similar pattern to the whole group comparison**.**

**Table 3:** Comparison of seroconversion and MAB in age and gender-matched cohorts

	**Measure**	**TP1** **2–4 w post** **2nd dose**	**TP2** **4 M post** **2nd dose**
**HD**	**% Seroconverted** ^a^MAB geo.mean (%inhibition)	**82%** 461.8 IU/ml (86%)	**79.3%** 152.2 IU/ml (61.40%)
**HC**	**% Seroconverted** ^a^MAB geo.mean (%inhibition)	**100%** 1940 IU/ml (97%)	**100%** 452.9 IU/ml (84.61%)
***P *value**	^***b***^***Chi sq*** ^***c***^*MWU* *(MWU)*	***P* < *0.001*** *P* < *0.001* *(P* < *0.001)*	***P* < *0.001*** *P* < *0.01* *(P* < *0.01)*

^a^MAB- mean/ median antibody level of the seropositives at each timepoint. Represented as geometric mean (geo.mean) in International Units (IU/ml) and as mean percentage inhibition (% inhibition)

^b^Chi sq test was performed to compare the % seroconverted at each timepoint

^c^Mann Whitney U (MWU) test was performed to compare the MAB levels of seropositives in both International units (IU/ml) and % inhibition. Comparison of % inhibition at each timepoint is shown within brackets. Significance at *p* < 0.05

### Factors associated with seropositivity and neutralizing antibody level in HD patients

**Fig. 4:**
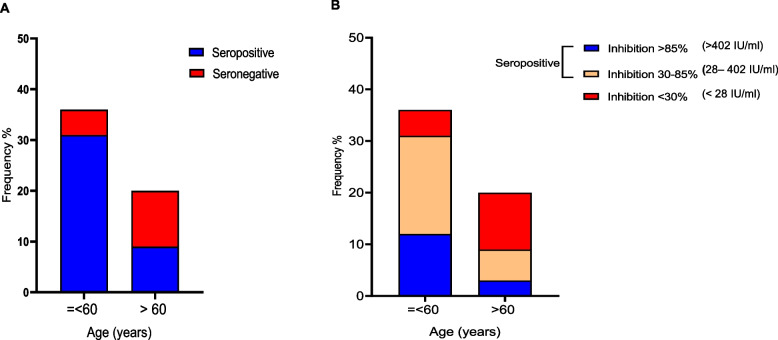
Factors associated with seropositivity and antibody level. Figure 4**A** shows the percentage of seropositive and seronegative individuals in each age group; ≤ 60 years and > 60 years. Four months following the second dose of AZD1222 (TP2), HD patients aged ≤ 60 years were significantly more likely to be seropositive than those > 60 years of age (chi sq df2, *p* < 0.001, OR: 7.58, CI:1.92–26.5). Figure **B** shows the percentage of seropositive and seronegative individuals in each % inhibition category; < 30%, 30%-85%, > 85% and in each age category; ≤ 60 years and > 60 years. HD patients aged < 60 years were more likely to maintain high antibody levels compared to older patients (chi sq df2, *p* < 0.005). Comparison between groups, Chi square for trend with Fisher’s exact test *p < 0.05, **p < 0.01, ***p < 0.001

At 2–4 weeks post 2^nd^ dose, seroconversion and nAB levels were not associated with patient factors. However at TP2-4 months post 2nd dose, HD patients aged < 60 years of age were significantly more likely to remain seropositive than those > 60 years of age (chi sq df_2,_ *p* < 0.001, OR: 7.58, CI:1.92–26.5)(Fig. 4A). with comparatively good antibody levels [47.5% (19/40) vs 15%(06/40), chi sq df_2,_ *p* < 0.005] (Fig. 4B). Other factors analyzed, including dialysis vintage (> 2yrs vs < 2yrs; *p* = 0,08), gender (*p* = 0.17), and presence of comorbidities including diabetes mellitus (*p* = 0.06) and dyslipidaemia (p0.32) were not associated with seroconversion status or nAB levels.

### Comparison of vaccine associated adverse events (AZD1222 and BNT162b2): HD vs HC)

**Fig. 5:**
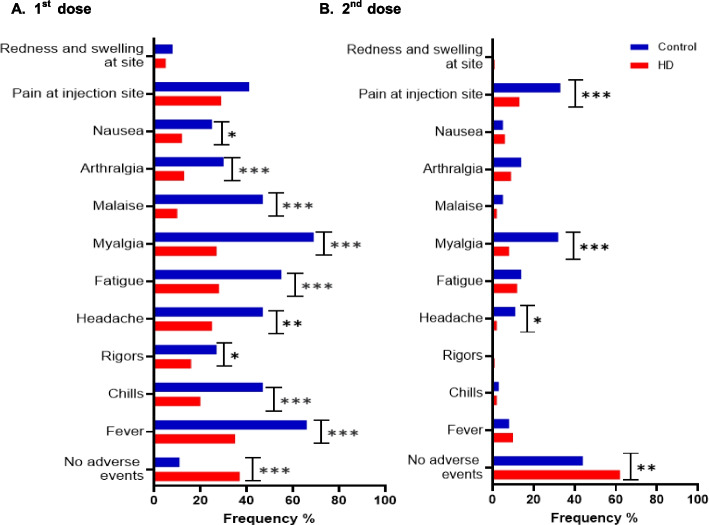
Adverse events following 2 doses of (ChAdOx1) Oxford Astrazeneca— AZD1222. The percentage of individuals in the healthy control (HC—blue) and haemodialysis (HD—red) cohorts who experienced adverse events following first and second doses of Oxford Astrazeneca -AZD1222 (ChAdOx1) are displayed as bar charts. Figure 5**A** shows the adverse events following the first dose. Figure 5**B** shows the adverse events following the second dose. Comparison between proportions Z test. *p < 0.05, **p < 0.01, ***p < 0.001

VAAE after the 1st dose of AZD1222 were reported by 88.89% of HCs and 62.16% of HD patients (Fig. 5). HD patients were significantly less likely to develop fever (chi sq df_2,_ *p* < 0.001), chills (*p* < 0.001), rigors (*p* < 0.001), headache (*p* < 0.001), fatigue (*p* < 0.001), myalgia (*p* < 0.001), malaise (*p* < 0.001), arthralgia (*p* < 0.05), nausea (*p* < 0.05). Following the 2nd dose of AZD1222 fewer individuals in both cohorts developed adverse effects (HC 55.88%, HD 37.33%). However, the HD patients were still less prone to pain at injection site (*p* < 0.001), myalgia (*p* < 0.001) and headache (*p* < 0.05). No VAAE in either group were noted after third dose administration of BNT162b2. Table 4 shows the summary of *p* values of each of the symptoms compared.

**Table 4:** Comparison of vaccine (ChAdOx1) Oxford Astrazeneca -AZD1222) related adverse events: HD VS HC

Adverse event	*P value*- 1^st^ dose	*P value*- 2^nd^ dose
No adverse events	< .001***	0.007**
Fever	< .001***	NS
Chills	< .001***	NS
Rigors	.041*	NS
Headache	0.001**	0.016*
Fatigue	< .001***	NS
Myalgia	< .001***	< .001***
Malaise	< .001***	NS
Arthralgia	0.002**	NS
Nausea	0.018*	NS
Pain at injection site	NS	< .001***
Redness and swelling at site	NS	NS

*p < 0.05, **p < 0.01, ***p < 0.001

## Discussion

We demonstrate seroconversion and SARS-CoV-2 nAB levels in patients on maintenance HD over an 18-month period following heterologous 3-dose vaccination (AZD1222—AZD1222—BNT162b2) compared to a health control group. To the best of our knowledge this is the first study to demonstrate nAB response to this vaccine regime in an HD cohort, along with 1-year follow-up data. Our results show significantly lower seroconversion with lower nAB levels in those who had seroconverted in the HD cohort compared to the healthy controls after two doses of AZD1222. After a third dose of heterologous mRNA vaccine BNT162b2, seroconversion and nAB levels increase significantly in both groups and at 1 year followup, both groups show similar levels of nAB while maintaining 100% seroconversion.

A recent study of UK registry data showed the improvement in all patient outcomes (hospitalization/ death etc.) for patients with kidney disease given this 3-dose combination (compared to 2-dose), clinically corroborating our evidence [37]. Similar studies on HD patients that were older than the control group, had patient groups with mean age ranging from 52–86 years [38–42], showing our HD cohort (mean age 54.1 years) to be comparatively young, though of similar age and gender distribution to other CKD patient groups studied in Sri Lanka [43].

Seroconversion rates vary between study populations depending on the vaccine used. Rates of 73%-92%, 2–4 weeks following 2 doses of Pfizer-BioNTech (BNT162b2) in HD patients (lower than healthy controls) have been shown in multiple studies [21, 38, 44–47]. The post 2nd dose seroconversion rate of HD patients, in our study (83.7% with MAB 450 IU/ml) was comparable with other work on similarly vaccinated HD cohorts (seroconversion 71.4% (MAB:345.1 BAU/ml) to 94.16% (MAB:1342 BAU/ml) [22, 48–51]. At four months post 2nd dose, the seroconversion rate in HD patients had dropped to 71.4% with MAB level of 235.4 IU/ml, similar to findings from Thailand where 2-dose AZD1222 in HD patients showed reducing seroconversion(92.06% to 82.26%) and MAB (anti-spike IgG, from 435.96 BAU/mL to 260.74 BAU/mL) in HD patients in 3 months post 2nd dose [51]. At 6 months post 2nd dose, though seropositivity in HDs dropped further to 66.7%, MAB level of patients who remained seropositive had not changed significantly as those with low antibody levels had become seronegative while those with moderate to high antibody appeared able to maintain levels. This is in contrast to other studies where the decay of nAB levels following 2 doses has been shown consistently in both healthy individuals [52, 53] and HD patients [50, 54–56]. These studies varied in terms of the patient demographics (mean age, dialysis vintage, prior infection status etc.) as well as the type of vaccine received. However, they consistently showed that 5–45% of HD patients became seronegative at 5–7 months post 2nd dose while antibody levels reduced by approximately 9–11 fold. These studies were, however, all conducted predominantly in developed settings (Netherlands [54], Switzerland [55], USA [57], and France [56]). In contrast, our study cohort faced the 3rd wave of COVID-19 predominantly due to the delta variant at the time, which accounted for the highest number of cases and deaths during the pandemic. Although no patients became symptomatic, asymptomatic infection resulting in persisting antibody levels causing this is possible.

Though the comparative efficacy of vaccines (BTN162B2 vs mRNA-1237 vs AZD1222) in HD patients is variable [55, 58–60] the increased efficacy seen after a 3rd dose for all disease outcomes is now clear [37]. It is also clear that a third dose is essential as nAB levels both for ancestral strain virus as well variants dropped rapidly by 6 months [50, 55].

Seropositivity and MAB level in both groups increased rapidly 2 weeks after the third dose (TP4) and showed further increase over the following one year with 100% seroconversion and comparably high MAB levels in both groups. Although there are fewer data available regarding the immune response to 3 dose-mixed vaccination in HD patients, several countries have studied the immune response to 3 doses of mRNA vaccines (BNT162b2 and/ or mRNA-1237) where seroconversion rates ranged between 95–98.5% [16, 29–31, 56, 57, 61] with increased antibody titres (SARS CoV-2 anti-spike IgG) that ranged between 9910 AU/ml and 26037AU/ml within 1–3 months of receiving the third booster dose. HD patients were shown to maintain seropositivity at 98.3% four months after the third dose, though MAB levels reduced [16]. Importantly, low to moderate levels of Omicron neutralization were seen in patients only after either 3rd dose vaccination or post COVID-19 infection [50, 57]. Further, while patients who had COVID-19 (after 2 previous doses) developed nAB levels similar to those who had 3rd dose vaccination (2200 -2500 IU/ml), those who had infection, had a 3.2 fold higher level of Omicron nAB compared to post vaccination individuals [57]. Studies also described HD patients who did not develop any anti-S IgG response after a third dose [29]. Non-response was associated with older age and immunosuppressive therapy whereas 40–70% of the 2nd dose non- or low-responders, had an effective antibody response after a 3rd dose [62, 63] Possibly, all HD patients in our cohort remained asymptomatic due to 3-dose vaccination generating some level of omicron neutralization, as the results show all 2-dose non-responders, responding to the 3rd dose.

Collectively, these findings highlight how vital the third boost to immunity is in HD patients, either by vaccine or by infection, as consistently high seroconversion rates, as well as durable antibody levels were produced. This is important in two respects. First, high antibody levels correlate with some neutralization of new variants, providing at least partial protection from an evolving virus. Data modeling by Cromer et al., showed that nAB to wild type SARS-CoV-2 was highly predictive of neutralization of variants of concern [64]. The current real world data on HD patient antibody dynamics corroborates this. The second is the duration of protection provided by a third dose. Available literature from Spain that followed up HD patients for 4 months post 3rd dose showing high (97%) seroconversion rates as well as more persistent antibody levels, with slower decay rates compared to post dose-2 [16]. Our work, though on a smaller scale shows similar results with high (100%) seroconversion and sustained antibody levels at 5 months post 3rd dose (1542 IU/ml). To our knowledge, we present the first data set that shows nAB levels at one year after 3rd dose in HD patients. We show consistently high levels of antibodies in almost all patients. The patients of this study were from a low-middle income setting, during a financial crisis and the follow-up period included the height of the delta variant dominant 3rd wave and omicron BA.5 dominant 4th wave. Community and healthcare associated exposure levels were likely high. Our results are consistent with what is known about post 3rd dose antibody responses, which are that they wane at a slower rate than those after the 2nd dose [53], even in HD patients [16]. It is also noted here that the BNT162b2 mRNA vaccine provided effective third dose immunity in a previously adenoviral vector vaccinated group of HD patients. This is also important with respect to the variable availability of vaccine platforms.

Our results are also interesting in light of recent evidence [65] that demonstrated nAB against wild type virus and delta variant after 2nd and 3rd dose had a decay half-life of approximately 60 days while the durability of nAB against three omicron subvariants (BA.1.1, BA.5, BA.2.12) was substantially better with decay half-lives of about 6 months. This study also showed that a 3rd dose of the original COVID-19 vaccine would broaden antibody responses against SARS-CoV, four other sarbecoviruses, and multiple SARS-CoV-2 strains. In conjunction with the strong evidence that natural infection provides a broader, more durable antibody repertoire against variants of concern, our results showing HD patients, who lived through delta and omicron waves, with substantial and durable antibody levels at one year post vaccination, provides hope that these vulnerable individuals will be at least partially protected from variants or even emerging sarbecoviruses to come. Further to this, there is evidence that a 4th dose of vaccine (triple BNT162b2 and fourth full-dose mRNA-1273) increased both the T cell responses as well as the omicron-specific antibody levels in both HD and healthy individuals [66].

At 4 months following the second dose both seropositivity and MAB of the HD cohort were associated with patient age < 60 years. Multiple studies have demonstrated that older age [19, 42, 44, 67, 68], lower lymphocyte count [19, 44, 67, 69] ow serum albumin levels [19, 69], longer dialysis vintage [69] use of corticosteroids/ immunosuppressive drugs [69, 70] lower vitamin-D levels, diabetes and history of cancer [44] are associated with poor antibody response while previous SARS CoV-2 infection [40, 68, 69] longer interval between vaccine doses [68], female gender [40] dialysis adequacy [55] were found to be associated with stronger antibody response. These findings are not consistent across studies [42, 48, 71] as the heterogeneity of findings are possibly due to the variability in age, type of dialysis, type of vaccine, background exposure levels of these cohorts.

In terms of VAAE, HD patients were less likely to experience most adverse events after 1st and 2nd doses compared to HCs. There were no patients or controls who developed significant adverse effects after the 3rd dose. These findings are consistent with other studies as well where HD patients did not develop adverse events following standard BNT162b2 vaccination [30] while some developed frequent but manageable side effects following heterologous AZD1222/ BNT162b2 compared to homologous BNT162b2 [72].

Overall, we report robust neutralizing antibody mediated immunity in HD patients, comparable to that of healthy individuals in response to a heterologous vaccine combination (AZD-1222- AZD-1222 – BNT 162b2) at 1 year post 3rd dose follow-up. With none of the patient cohort developing documented COVID-19, and available evidence pointing to broadly neutralizing, durable immunity being generated with repeated exposure (whether vaccine or infection or combination induced), our data suggests that HD patients who received 3-doses of COVID-19 vaccines have higher levels of long term protection, and broader protection than was perhaps initially estimated.

## Limitations

This study has several limitations. The HD patients and controls were not age matched. Though matched subgroup comparisons were done for initial timepoints, as study dropout rates increased in patients, subsequent matched analysis could not be done. However, even with a significantly older cohort of patients, seroconversion rates and antibody levels were similar to healthy individuals after 3rd dose vaccination indicating a robust response. All timepoints could not be sampled from both HD and HC groups throughout follow-up for logistic reasons which included sampling limitations due to lack of test kits as well as drop-outs of HD patients especially during the height of the 3rd wave of the pandemic in Sri Lanka. Ideally, we would have continued to follow-up the HD patients who did not receive the 3rd dose for longitudinal comparison, but this was not feasible at that time.

Based on seroconversion statistics, the required sample size could not be calculated accurately as all HCs in this study seroconverted. However the sample size is adequate for comparison of this outcome at TP1/2 if the probability of seroconversion in HCs is taken as 0.999 with an unequal sample size of 2:1 HD:HC. Sample sizes at subsequent timepoints for robust comparison are difficult to ascertain particularly as seroconversion rates reach 100% in both cohorts. Comparison of MAB (geo. mean and % inhibition) show greater differences between groups and based on sample size calculations for mean comparisons, sample of 17–20 are adequate. However, as non-parametric tests are used, approximately 23 per group (15% increase as a rule of thumb) would be required. This sample number is therefore adequate for the initial comparisons but remains below optimum for the latter TP5 and TP6 comparisons. Follow-up of the larger group and including a completely age and gender match control group would have been ideal. However, limitations in testing and logistic realities required curtailing testing. Though sample numbers towards the latter groups reduced, the consistency of the results across all samples tested indicates that the findings are unlikely to be spurious or due to random chance, which are the main concerns. Further evaluation in larger cohorts would be needed.

Samples with very high nAB levels required dilution re-testing which was not feasible. The samples tested gave results ranging from 97.6–98.4% showing high consistency in percentage inhibition value while the converted unit values ranged from 3061–29633 IU/ml providing a range of scale of nAB in high responders. Obtaining exact values by dilution testing would alter only the geometric mean AB IU/ml results. As comparison of these antibody unit levels was done with rank tests, there is no effect of the size of the value. We therefore presume minimal effect on comparisons at TP1 and TP2 (where 4–9% of samples required dilution). (TP 4 and 5 had no samples requiring dilution). While is it possible that HC had significantly higher nAB levels (IU/ml) than HDs particularly at TP6, where 19/30 sample required dilution testing (HC-11, HD-8; four dilution tested samples show HD nAB levels 3064 – 10,604 IU/ml and HC nAB level – 29,633 IU/ml), the results showing persistent high nAB levels remains robust. As we are able to show durable response to heterologous vaccination up to 1 year post third dose, we believe this lack of dilution testing does not affect this conclusion. As we did not perform anti-N protein (viral neucleocapsid) Ig testing, we are unable to differentiate how many of the study cohorts had COVID-19, which would account for some part of the antibody response seen.

## Conclusion

In conclusion, HD patients respond comparatively poorly to 2- dose vaccination of AZD-1222 – AZD-1222. A third dose of BNT162b2 vaccine significantly improves the seroconversion rate and the MAB of HD patients, comparable to the HCs with a durable nAB response lasting at least 1 year post vaccination.

### Supplementary Information

Additional file 1.

Additional file 2.

## Data Availability

The data supporting the findings of this study are available from the corresponding author on request. As analysis of the data has not yet been completed, these data are not yet being made available in public repositories. No additional data or code were used in generation of results.

## Abbreviations

CKD: Chronic Kidney Disease
EC: Ethical Review Committee
HC: Healthy Control
HD: Haemodialysis
IQR: Interquartile Range
MAB: Mean Antibody level
mRNA: Messenger Riboneucleic acid
MWU: Mann Whitney U
nAB: Neutralizing antibodies
OR: Odds Ratio
SAGE: Strategic Advisory Group of Experts
SD: Standard Deviation
TP: Timepoint
VAAE: Vaccine Associated Adverse Events
WHO: World Health Organization

## References

[1] Salerno S, Messana JM, Gremel GW, Dahlerus C, Hirth RA, Han P (2021). COVID-19 Risk Factors and Mortality Outcomes among Medicare Patients Receiving Long-term Dialysis. JAMA Netw Open.

[2] Couchoud C, Bayer F, Ayav C, Béchade C, Brunet P, Chantrel F (2020). Low incidence of SARS-CoV-2, risk factors of mortality and the course of illness in the French national cohort of dialysis patients. Kidney Int.

[3] Ng JH, Hirsch JS, Wanchoo R, Sachdeva M, Sakhiya V, Hong S (2020). Outcomes of patients with end-stage kidney disease hospitalized with COVID-19. Kidney Int.

[4] Alberici F, Delbarba E, Manenti C, Econimo L, Valerio F, Pola A (2020). A report from the Brescia Renal COVID Task Force on the clinical characteristics and short-term outcome of hemodialysis patients with SARS-CoV-2 infection. Kidney Int.

[5] Hilbrands LB, Duivenvoorden R, Vart P, Franssen CFM, Hemmelder MH, Jager KJ (2020). COVID-19-related mortality in kidney transplant and dialysis patients: Results of the ERACODA collaboration. Nephrol Dial Transplant.

[6] Jager KJ, Kramer A, Chesnaye NC, Couchoud C, Sánchez-Álvarez JE, Garneata L (2020). Results from the ERA-EDTA Registry indicate a high mortality due to COVID-19 in dialysis patients and kidney transplant recipients across Europe. Kidney Int.

[7] Kho MML, Reinders MEJ, Baan CC, Baarle D Van, Bemelman FJ, Diavatopoulos DA (2021). The RECOVAC IR study : the immune response and safety of the mRNA-1273 COVID-19 vaccine in patients with chronic kidney disease , on dialysis or living with a kidney transplant. Nephrol Dial Transplant.

[8] Francis A, Baigent C, Ikizler TA, Cockwell P, Jha V. The urgent need to vaccinate dialysis patients against severe acute respiratory syndrome coronavirus 2: a call to action. Vol. 99, Kidney International. Elsevier B.V.; 2021. 791–3. 10.1016/j.kint.2021.02.003 PMC7879104 33582109

[9] Simon B, Rubey H, Treipl A, Gromann M, Hemedi B, Zehetmayer S, et al. Haemodialysis patients show a highly diminished antibody response after COVID-19 mRNA vaccination compared with healthy controls. 2021;2204–6. 10.1093/ndt/gfab179 PMC8194560 33999200

[10] de Meester J, de Bacquer D, Naesens M, Meijers B, Couttenye MM, de Vriese AS (2021). Incidence, characteristics, and outcome of COVID-19 in adults on kidney replacement therapy: A regionwide registry study. J Am Soc Nephrol.

[11] Williamson EJ, Walker AJ, Bhaskaran K, Bacon S, Bates C, Morton CE, et al. Factors associated with COVID-19-related death using OpenSAFELY. Nature [Internet]. 2020;584(7821):430–6. Available from: 10.1038/s41586-020-2521-4. 10.1038/s41586-020-2521-4 PMC7611074 32640463

[12] Semenzato L, Botton J, Drouin J, Cuenot F, Dray-Spira R, Weill A, et al. Chronic diseases, health conditions and risk of COVID-19-related hospitalization and in-hospital mortality during the first wave of the epidemic in France: a cohort study of 66 million people. Lancet Reg Heal - Eur. 2021;8. 10.1016/j.lanepe.2021.100158 PMC8282330 34308411

[13] Stevens CE, Alter HJ, Taylor PE, Zang EA, Harley EJ, Szmuness W. Hepatitis B Vaccine in Patients Receiving Hemodialysis. N Engl J Med. 1984;311(8):496–501.Available from: 10.1056/NEJM198408233110803. 10.1056/NEJM198408233110803 6235453

[14] Fuchshuber A, Kuhnemund O, Keuth B, Lutticken R, Michalk D, Querfeld U. Nephrology Dialysis Transplantation Pneumococcal vaccine in children and young adults with chronic renal disease. 1996;468–73. 8671817

[15] Trial L term HAO label. Immunogenicity of a Standard Trivalent Influenza Vaccine in Patients on. YAJKD [Internet]. 2009;54(1):77–85. Available from: 10.1053/j.ajkd.2008.11.032. 10.1053/j.ajkd.2008.11.032 19339089

[16] Gallego-valcarce E, Shabaka A, Leon-poo M, Gruss E, Acedo-sanz JM, Cordón A, et al. Humoral Response Following Triple Dose of mRNA Vaccines Against SARS-CoV-2 in Hemodialysis Patients : Results After 1 Year of. 2022;9:1–12. 10.3389/fmed.2022.927546 PMC9314744 35903310

[17] Peiyao R, Mengjie Y, Xiaogang S, Wenfang H. Immunogenicity and safety of SARS-CoV- vaccine in hemodialysis patients : A systematic review and. 10.3389/fpubh.2022.951096 PMC9539993 36211647

[18] Galmiche S, Binh L, Nguyen L, Tartour E, Lamballerie X De, Wittkop L, et al. Immunological and clinical ef fi cacy of COVID-19 vaccines in immunocompromised populations : a systematic review. Clin Microbiol Infect [Internet]. 2022;28(2):163–77. Available from: 10.1016/j.cmi.2021.09.036. 10.1016/j.cmi.2021.09.036 PMC8595936 35020589

[19] Angel-korman A, Peres E, Bryk G, Lustig Y, Indenbaum V, Amit S (2022). Diminished and waning immunity to COVID-19 vaccination among hemodialysis patients in Israel : the case for a third vaccine dose. Clin Kidney J..

[20] Espi M, Charmetant X, Barba T, Mathieu C, Pelletier C, Koppe L, et al. No Title. 2020.

[21] Falahi S. Immunogenicity of COVID ‐ 19 mRNA vaccines in hemodialysis patients : Systematic review and meta ‐ analysis. 2022. 10.1002/hsr2.874 PMC9528953 36210877

[22] Wang H hao, Wu J ling, Chang M yu, Wu H mian, Ho L chun, Chi P jui, et al. Antibody Response and Adverse Events of AZD1222 COVID-19 Vaccination in Patients Undergoing Dialysis : A Prospective Cohort Study. 2022; 10.3390/vaccines10091460 PMC9501178 36146538

[23] Garcia P, Anand S, Han J, Montez-Rath ME, Sun S, Shang T (2022). COVID-19 Vaccine Type and Humoral Immune Response in Patients Receiving Dialysis. J Am Soc Nephrol.

[24] Murt A (2022). Antibody responses to the SARS-CoV-2 vaccines in hemodialysis patients : Is inactivated vaccine effective ?. Ther Apher Dial..

[25] Anand S, Montez-Rath ME, Han J, Garcia P, Cadden L, Hunsader P, et al. SARS-CoV-2 Vaccine Antibody Response and Breakthrough Infection in Patients Receiving Dialysis. Ann Intern Med [Internet]. 2021;175(3):371–8. Available from: 10.7326/M21-4176. 10.7326/M21-4176 PMC8722718 34904856

[26] Hsu CM, Weiner DE, Manley HJ, Aweh GN, Ladik V, Frament J (2022). Seroresponse to SARS-CoV-2 Vaccines among Maintenance Dialysis Patients over 6 Months. Clin J Am Soc Nephrol.

[27] Strategic Advisory Group of Experts (SAGE), Parker E, Desai S, Wilder-Smith A, Hombach J, Marti M. Interim recommendations for an extended primary series with an additional vaccine dose for COVID-19 vaccination in immunocompromised persons. 2023.

[28] Rodr D, Cuadrado E, Bedini L. Humoral Response after Three Doses of mRNA-1273 or BNT162b2 SARS-CoV-2 Vaccines in Hemodialysis Patients. 2022; 10.3390/vaccines10040522 PMC9030003 35455271

[29] Melin J, Svensson MK, Albinsson B, Winqvist O, Pauksens K. A third dose SARS ‑ CoV ‑ 2 BNT162b2 mRNA vaccine results in improved immune response in hemodialysis patients. 2022; 10.48101/ujms.v127.8959 PMC9602199 36337280

[30] Shashar M (2022). Humoral Response to Pfizer BNT162b2 Vaccine Booster in Maintenance Hemodialysis Patients. Am J Nephrol..

[31] Verdier JF, Boyer S, Chalmin F, Jeribi A, Egasse C, Maggi MF, et al. Response to three doses of the Pfizer/BioNTech BNT162b2 COVID-19 vaccine: a retrospective study of a cohort of haemodialysis patients in France. BMC Nephrol [Internet]. 2022;23(1):1–13. Available from: 10.1186/s12882-022-02751-5. 10.1186/s12882-022-02751-5 PMC9116059 35585512

[32] Wijewickrama ES, Herath N (2022). Global Dialysis Perspective: Sri Lanka. Kidney360.

[33] Tan CW, Chia WN, Qin X, Liu P, Chen MIC, Tiu C, et al. A SARS-CoV-2 surrogate virus neutralization test based on antibody-mediated blockage of ACE2–spike protein–protein interaction. Nat Biotechnol [Internet]. 2020;38(9):1073–8. Available from: 10.1038/s41587-020-0631-z. 10.1038/s41587-020-0631-z 32704169

[34] Kristiansen PA, Page M, Bernasconi V, Mattiuzzo G, Dull P, Makar K, et al. WHO International Standard for anti-SARS-CoV-2 immunoglobulin. Lancet [Internet]. 2021;397(10282):1347–8. Available from: 10.1016/S0140-6736(21)00527-4. 10.1016/S0140-6736(21)00527-4 PMC7987302 33770519

[35] Zhu F, Althaus T, Tan CW, Costantini A, Chia WN, Van Vinh CN (2022). WHO international standard for SARS-CoV-2 antibodies to determine markers of protection. The Lancet Microbe.

[36] Lee SW (2022). Regression analysis for continuous independent variables in medical research: statistical standard and guideline of Life Cycle Committee. Life Cycle.

[37] Parker EPK, Horne EMF, Hulme WJ, Tazare J, Zheng B, Carr EJ, et al. Comparative effectiveness of two- and three-dose COVID-19 vaccination schedules involving AZD1222 and BNT162b2 in people with kidney disease: a linked OpenSAFELY and UK Renal Registry cohort study. Lancet Reg Heal - Eur [Internet]. 2023;30:100636. Available from: 10.1016/j.lanepe.2023.100636. 10.1016/j.lanepe.2023.100636 PMC10155829 37363796

[38] Kitamura M, Takazono T, Yamamoto K, Harada T, Funakoshi S, Mukae H, et al. Low humoral immune response to the BNT162b2 vaccine against COVID-19 in nursing home residents undergoing hemodialysis: a case–control observational study. Ren Replace Ther [Internet]. 2022;8(1):1–9. Available from: 10.1186/s41100-022-00397-5. 10.1186/s41100-022-00397-5 PMC8924726 35308296

[39] Bai S, Dhrolia M, Qureshi H, Qureshi R, Nasir K, Ahmad A (2022). Comparison of COVID-19 Inactivated Virus Vaccine Immunogenicity Between Healthy Individuals and Patients on Hemodialysis: A Single-Center Study From Pakistan. Cureus.

[40] Füessl L, Lau T, Lean I, Hasmann S, Riedl B, Arend FM, et al. Diminished Short- and Long-Term Antibody Response after SARS-CoV-2 Vaccination in Hemodialysis Patients. 2022;1–10. 10.3390/vaccines10040605 PMC9031197 35455353

[41] Behrens GMN, Cossmann A, Stankov MV (2020). Strategic Anti-SARS-CoV-2 Serology Testing in a Low Prevalence Setting : The COVID-19 Contact ( CoCo ) Study in Healthcare Professionals. Infect Dis Ther..

[42] Cheng CY, Hsiao SH, Fang TC, Lin YC, Wang JCC, Hung CS (2022). SARS-CoV2 antibody response after a third dose of heterologous ChAdOx1 nCoV-19 and Moderna vaccine in chronic dialysis patients. J Infect.

[43] Ranasinghe AV, Kumara GWGP, Karunarathna RH, De Silva AP, Sachintani KGD, Gunawardena JMCN (2019). The incidence, prevalence and trends of Chronic Kidney Disease and Chronic Kidney Disease of uncertain aetiology (CKDu) in the North Central Province of Sri Lanka: An analysis of 30,566 patients. BMC Nephrol.

[44] Ben-Dov IZ, Oster Y, Tzukert K, Alster T, Bader R, Israeli R, et al. Impact of tozinameran (BNT162b2) mRNA vaccine on kidney transplant and chronic dialysis patients: 3–5 months follow-up. J Nephrol [Internet]. 2022;35(1):153–64. Available from: 10.1007/s40620-021-01210-y 10.1007/s40620-021-01210-y PMC8731189 34988942

[45] Speer C, Goth D, Benning L, Buylaert M, Schaier M, Grenz J (2021). Early humoral responses of hemodialysis patients after covid-19 vaccination with bnt162b2. Clin J Am Soc Nephrol.

[46] Al-Muhaiteeb A, AlSahow A, Al-Yousef A, AlHelal B, Alrajab H, Bahbahani Y (2022). Response to and outcomes of the Pfizer BNT162B2 vaccine in hemodialysis patients—A prospective observational study. Hemodial Int.

[47] Falahi S, Sayyadi H, Kenarkoohi A (2022). Immunogenicity of COVID-19 mRNA vaccines in hemodialysis patients: Systematic review and meta-analysis. Heal Sci Reports..

[48] Affeldt P, Koehler FC, Brensing KA, Adam V, Burian J, Butt L, et al. Immune Responses to SARS-CoV-2 Infection and Vaccination in Dialysis Patients and Kidney Transplant Recipients. 2022;1–14. 10.3390/microorganisms10010004 PMC8779774 35056453

[49] Cheng CY, Fang TC, Liao HW, Chen TH, Chang JH, Lin YC, et al. The Humoral Immune Response of the ChAdOx1 nCoV-19 Vaccine in Maintenance Dialysis Patients without Prior COVID-19 Infection. Vaccines. 2022;10(2). 10.3390/vaccines10020338 PMC8879203 35214797

[50] Ling TC, Chen PL, Li NY, Ko WC, Sun CY, Chao JY (2023). Trajectory of Humoral Responses to Two Doses of ChAdOx1 nCoV-19 Vaccination in Patients Receiving Maintenance Hemodialysis. Microbiol Spectr.

[51] Prasithsirikul W, Nopsopon T, Phutrakool P, Suwanwattana P, Kantagowit P, Pongpirul W (2022). ChAdOx1 nCoV-19 Immunogenicity and Immunological Response Following COVID-19 Infection in Patients Receiving Maintenance Hemodialysis. Vaccines..

[52] Glück V, Grobecker S, Köstler J, Tydykov L, Bertok M, Weidlich T, et al. Immunity after COVID-19 and vaccination: follow-up study over 1 year among medical personnel. Infection [Internet]. 2022;50(2):439–46. Available from: 10.1007/s15010-021-01703-9. 10.1007/s15010-021-01703-9 PMC8475821 34562263

[53] Gilboa M, Regev-Yochay G, Mandelboim M, Indenbaum V, Asraf K, Fluss R (2022). Durability of Immune Response after COVID-19 Booster Vaccination and Association with COVID-19 Omicron Infection. JAMA Netw Open.

[54] Sanders JSF, Messchendorp AL, De Vries RD, Baan CC, Van Baarle D, Van Binnendijk R, et al. Antibody and T-Cell Responses 6 Months after Coronavirus Disease 2019 Messenger RNA-1273 Vaccination in Patients with Chronic Kidney Disease, on Dialysis, or Living with a Kidney Transplant. Clin Infect Dis [Internet]. 2023;76(3):E188--E199. Available from: 10.1093/cid/ciac557. 10.1093/cid/ciac557 PMC9278186 35796536

[55] Bassi J, Giannini O, Silacci-Fregni C, Pertusini L, Hitz P, Terrot T (2022). Poor neutralization and rapid decay of antibodies to SARS-CoV-2 variants in vaccinated dialysis patients. PLoS One..

[56] Espi M, Charmetant X, Mathieu C, Lalande A, Decimo D, Koppe L, et al. Rapid Waning of Immune Memory Against SARS-CoV-2 in Maintenance Hemodialysis Patients After mRNA Vaccination and Impact of a Booster Dose. Kidney Int Reports [Internet]. 2023;8(4):907–11. Available from: 10.1016/j.ekir.2023.01.004. 10.1016/j.ekir.2023.01.004 PMC9827668 36644712

[57] Wang X, Han M, Fuentes LR, Thwin O, Grobe N, Wang K (2022). SARS-CoV-2 neutralizing antibody response after three doses of mRNA1273 vaccine and COVID-19 in hemodialysis patients. Front Nephrol.

[58] Sanders JSF, Bemelman FJ, Messchendorp AL, Baan CC, Van Baarle D, Van Binnendijk R (2022). The RECOVAC Immune-response Study: The Immunogenicity, Tolerability, and Safety of COVID-19 Vaccination in Patients With Chronic Kidney Disease, on Dialysis, or Living With a Kidney Transplant. Transplantation.

[59] Chinnadurai R, Wu HHL, Cox E, Moore J, Clough T, Lamerton E (2022). Humoral Response in Hemodialysis Patients Following COVID-19 Vaccination and Breakthrough Infections during Delta and Omicron Variant Predominance. Vaccines..

[60] Carr EJ, Wu M, Harvey R, Wall EC, Kelly G, Hussain S, et al. Neutralising antibodies after COVID-19 vaccination in UK haemodialysis patients. Vol. 398, Lancet (London, England). 2021. 1038–41. 10.1016/S0140-6736(21)01854-7 PMC8360704 34391504

[61] Agur T, Zingerman B, Ben-Dor N, Alkeesh W, Steinmetz T, Rachamimov R, et al. Humoral Response to the Third Dose of BNT162b2 COVID-19 Vaccine among Hemodialysis Patients. Nephron. 2022; 10.1159/000525519 PMC9747736 35896080

[62] Tillmann F peter, Figiel L, Ricken J, Still H, Korte C, Plassmann G, et al. Evolution of SARS-CoV-2-Neutralizing Antibodies after Two Standard Dose Vaccinations , Risk Factors for Non-Response and Effect of a Third Dose Booster Vaccination in Non-Responders on Hemodialysis : A Prospective Multi-Centre Cohort Study. 2021; 10.3390/jcm10215113 PMC8584296 34768631

[63] Robert T, Lano G, Giot M, Fourié T, Lamballeri  X De, Jehel O (2022). Humoral response after SARS-CoV-2 vaccination in patients undergoing maintenance haemodialysis : loss of immunity , third dose and non-responders. Nephrol Dial Transplant.

[64] Cromer D, Steain M, Reynaldi A, Schlub TE, Wheatley AK, Juno JA, et al. Neutralising antibody titres as predictors of protection against SARS-CoV-2 variants and the impact of boosting: a meta-analysis. The Lancet Microbe [Internet]. 2022;3(1):e52--e61. Available from: 10.1016/S2666-5247(21)00267-6. 10.1016/S2666-5247(21)00267-6 PMC8592563 34806056

[65] Nair MS, Ribeiro RM, Wang M, Bowen AD, Liu L, Guo Y, et al. Changes in serum-neutralizing antibody potency and breadth post-SARS-CoV-2 mRNA vaccine boost. iScience [Internet]. 2023;26(4):106345. Available from: 10.1016/j.isci.2023.106345. 10.1016/j.isci.2023.106345 PMC9987605 36925721

[66] Dopfer- A, Dulovic A, Affiliations A. Longitudinal cellular and humoral immune responses after triple BNT162b2 and fourth full-dose mRNA-1273 vaccination in haemodialysis patients. 2022;49(0):0–16. 10.3389/fimmu.2022.1004045 PMC9582343 36275672

[67] Grupper A, Sharon N, Finn T, Cohen R, Israel M, Agbaria A, et al. Humoral Response to the Pfizer BNT162b2 Vaccine in Patients Undergoing Maintenance Hemodialysis. Clin J Am Soc Nephrol. 2021;CJN.03500321. 10.2215/CJN.03500321 PMC8425628 33824157

[68] Haarhaus M, Duhanes M, Leševic N, Matei B, Ramsauer B, Da Silva RR (2022). Improved immunologic response to COVID-19 vaccine with prolonged dosing interval in haemodialysis patients. Scand J Immunol.

[69] Van Praet J, Reynders M, De Bacquer D, Viaene L, Schoutteten MK, Caluwé R (2021). Predictors and dynamics of the humoral and cellular immune response to SARS-CoV-2 mRNA vaccines in hemodialysis patients: A multicenter observational study. J Am Soc Nephrol.

[70] Galmiche S, Binh L, Nguyen L, Tartour E, Lamballerie X De, Wittkop L, et al. Since January 2020 Elsevier has created a COVID-19 resource centre with free information in English and Mandarin on the novel coronavirus COVID- 19 . The COVID-19 resource centre is hosted on Elsevier Connect , the company ’ s public news and information . 2020.

[71] Attias P, Sakhi H, Rieu P, Soorkia A, Assayag D, Bouhroum S, et al. Antibody response to the BNT162b2 vaccine in maintenance hemodialysis patients. Vol. 99, Kidney international. 2021. 1490–2. 10.1016/j.kint.2021.04.009 PMC8055946 33887317

[72] Haase M, Lesny P, Anderson M, Cloherty G, Stec M, Haase-Fielitz A, et al. Humoral immunogenicity and tolerability of heterologous ChAd/BNT compared with homologous BNT/BNT and ChAd/ChAd SARS-CoV-2 vaccination in hemodialysis patients: A multicenter prospective observational study. J Nephrol [Internet]. 2022;35(5):1467–78. Available from: 10.1007/s40620-022-01247-7. 10.1007/s40620-022-01247-7 PMC8792133 35084719

