# Supplementary material for: 18-month longitudinal SARS COV-2 neutralizing antibody dynamics in haemodialysis patients receiving heterologous 3-dose vaccination (AZD-1222- AZD-1222- BNT162b2) in a lower middle income setting
Source: BMC Nephrol. 2024 May 22;25:176. doi: 10.1186/s12882-024-03599-7 (PMC11112903; doi:10.1186/s12882-024-03599-7)
Supplement: Supplementary file 1 — Additional file 1. [file 12882_2024_3599_MOESM1_ESM.pdf]

# Additional figure 1

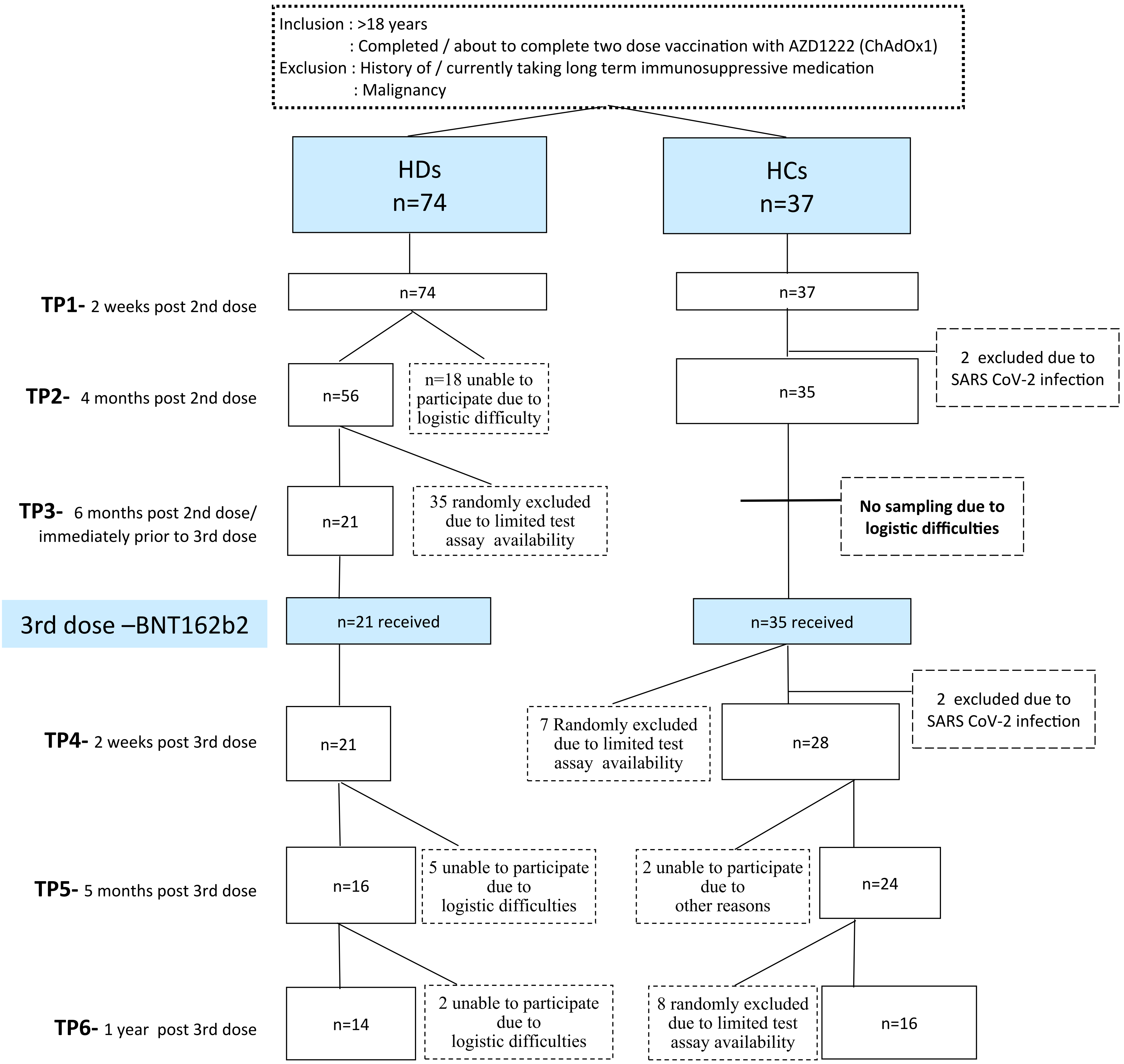


**Additional figure 1. Participant recruitment in HD and HC cohorts.** The flow diagram shows the number of participants recruited, excluded and lost to follow-up at each time point.
