# Supplementary material for: 18-month longitudinal SARS COV-2 neutralizing antibody dynamics in haemodialysis patients receiving heterologous 3-dose vaccination (AZD-1222- AZD-1222- BNT162b2) in a lower middle income setting
Source: BMC Nephrol. 2024 May 22;25:176. doi: 10.1186/s12882-024-03599-7 (PMC11112903; doi:10.1186/s12882-024-03599-7)
Supplement: Supplementary file 2 — Additional file 2. [file 12882_2024_3599_MOESM2_ESM.pdf]

# Additional figure 2

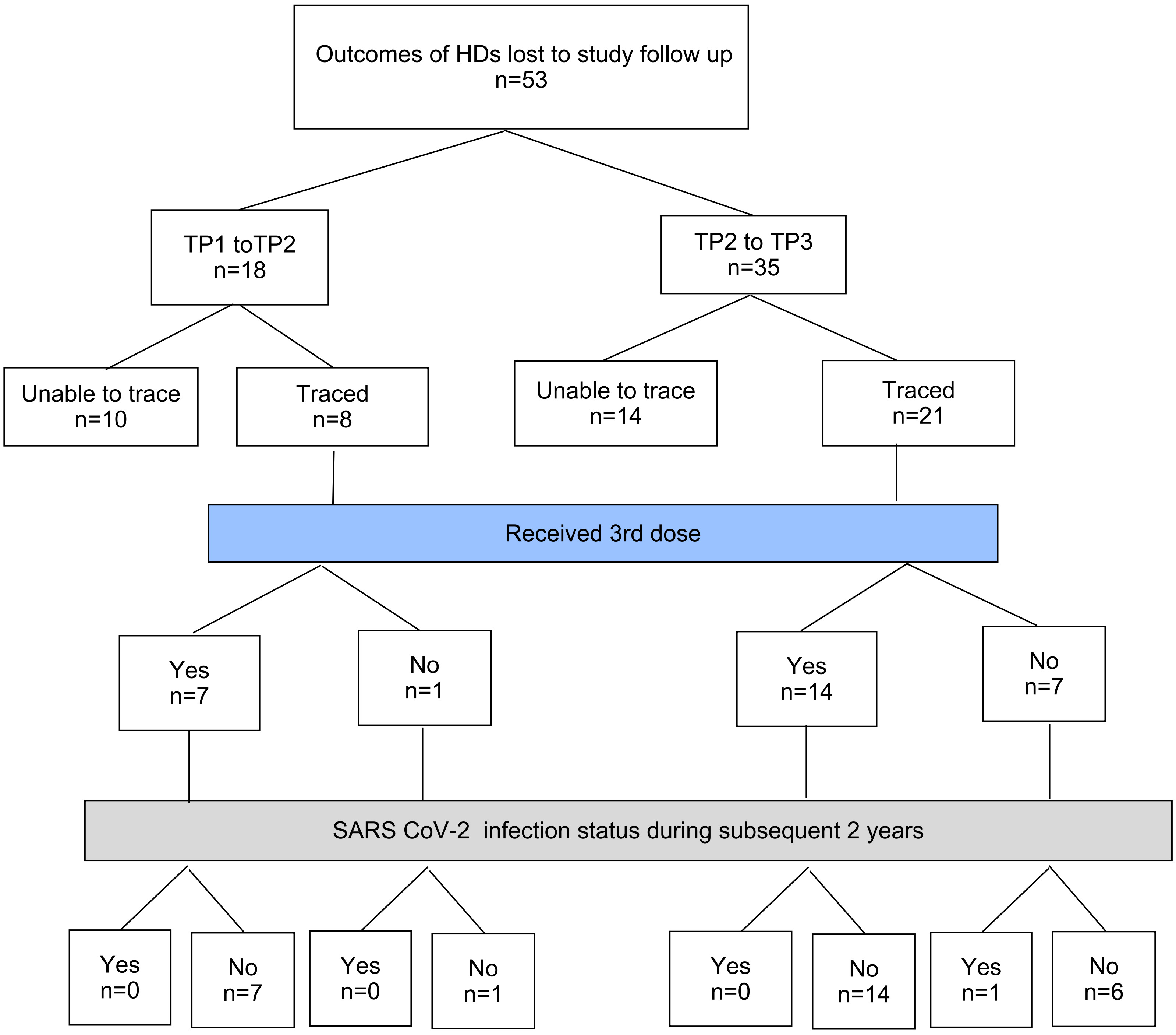


**Additional figure 2. Post-study tracing of individuals lost to follow-up in study period (HD cohort).** The flow diagram shows the number of study participants in the HD cohort who were lost to follow-up during the study period at various time points who were traceable/ untraceable at the time of publication of this paper with outcomes. 21/20 traced individuals received the 3rd dose while 8/29 did not. Only one patient has a documented COVID-19 infection among this group.
